# Reversion Mosaicism in Primary Immunodeficiency Diseases

**DOI:** 10.3389/fimmu.2021.783022

**Published:** 2021-11-16

**Authors:** Hanae Miyazawa, Taizo Wada

**Affiliations:** Department of Pediatrics, School of Medicine, Institute of Medical, Pharmaceutical and Health Sciences, Kanazawa University, Kanazawa, Japan

**Keywords:** reversion, reversion mosaicism, somatic reversion, primary immunodeficiency diseases, selective advantage, gene therapy

## Abstract

Reversion mosaicism has been reported in an increasing number of genetic disorders including primary immunodeficiency diseases. Several mechanisms can mediate somatic reversion of inherited mutations. Back mutations restore wild-type sequences, whereas second-site mutations result in compensatory changes. In addition, intragenic recombination, chromosomal deletions, and copy-neutral loss of heterozygosity have been demonstrated in mosaic individuals. Revertant cells that have regained wild-type function may be associated with milder disease phenotypes in some immunodeficient patients with reversion mosaicism. Revertant cells can also be responsible for immune dysregulation. Studies identifying a large variety of genetic changes in the same individual further support a frequent occurrence of reversion mosaicism in primary immunodeficiency diseases. This phenomenon also provides unique opportunities to evaluate the biological effects of restored gene expression in different cell lineages. In this paper, we review the recent findings of reversion mosaicism in primary immunodeficiency diseases and discuss its clinical implications.

## Introduction

**Figure 1:**
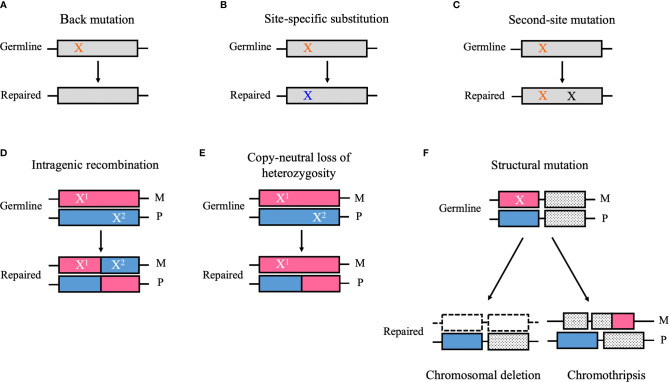
Schematic diagrams of the reversion mechanism. **(A)** A germ line point mutation (orange “X”) is changed to the wild-type sequence (i.e., back mutation). **(B)** A germ line point mutation substitutes for a nucleotide other than the wild-type sequence (blue “X”), which restores the original amino acid sequence or results in the alternation to a less deleterious amino acid than in the original germline mutation (i.e., site-specific substitution). **(C)** A mutation that occurs at a different site from the germline mutation but within the coding or noncoding regions of the same gene (black “X”) can cause a compensatory change that abrogates the deleterious effect of the germline mutation (i.e., second-site mutation). **(D)** Intragenic recombination can lead to reversion through the generation of a wild-type allele, whereas the other allele carries both germline mutations (i.e., X^1^ and X^2^). **(E)** Copy-neutral loss of heterozygosity can eliminate the chromosomal region encompassing the germline mutation and replace it a copy of the wild-type chromosome from the other parent. **(F)** A structural mutation such as chromosomal deletion and chromothripsis can abrogate a deleterious gain-of-function mutation (“X”) by modifying the chromosomal structure. M, maternal allele; P, paternal allele. **(A)** Back mutation, **(B)** Site-specific substitution, **(C)** Second-site mutation, **(D)** Intragenic recombination, **(E)** Copy-neutral loss of heterozygosity, **(F)** Structural mutation.

Genetic mosaicism refers to an individual who has developed from a unique zygote but carries two or more cell types with different genotypes (1). This phenomenon is derived from postzygotic mutations, which can occur during embryonic development or during postnatal life (1). Based on the tissue distributions, genetic mosaicism is categorized into three types: gonadal mosaicism, gonosomal mosaicism, and somatic mosaicism (1). In gonadal and gonosomal mosaicism, postzygotic mutations affect the germline cells; therefore, mutant alleles can be transmitted to the offspring (1). However, in somatic mosaicism, postzygotic mutations occur only in somatic cells and may cause a disease related to the mutated gene, but they are not transmitted to the offspring (1). Reversion mosaicism refers to somatic mosaicism due to a reversion to normal of an inherited pathogenic mutation (2). In reversion mosaicism, reversion mutations partially or fully restore the effect of the primary disease-causing variant (2). The most common and simplest type of reversion is a true back mutation, which refers to the reversion of the germline mutation site to the wild-type sequence (3) (**Figure 1A**). As an alternative, a site-specific substitution is a nucleotide substitution at the specific germline mutation site, which restores the original amino acid sequence or the alternation to a less deleterious amino acid than in the original germline mutation (3) (**Figure 1B**). Reversion mutation also results from second-site mutation, which occurs at a different site from the germline mutation but within the coding or noncoding regions of the same gene, and results in a compensatory change that abrogates the deleterious effect of the germline mutation (3) (**Figure 1C**). In autosomal recessive disorders caused by compound heterozygous mutations, intragenic recombination can lead to reversion through the generation of a wild-type allele with the other allele carrying both germline mutations (3) (**Figure 1D**). Copy-neutral loss of heterozygosity (CN-LOH) can eliminate the chromosomal region encompassing the germline mutation and replace it with a copy of the wild-type chromosome from the other parent (3) (**Figure 1E**). Furthermore, in autosomal dominant disorders caused by gain-of-function mutations, the elimination of a dominant germline mutation can be achieved by aneuploidy resulting from chromosomal structural mutations such as chromosomal deletion and chromothripsis (3) (**Figure 1F**).

Reversion mosaicism has been described in several disorders that affect the hematopoietic and nonhematopoietic systems such as Bloom syndrome, Fanconi anemia, dyskeratosis congenita, tyrosinemia, and epidermolysis bullosa (3, 4). Reversion mosaicism has frequently been found in diseases that affect highly generating tissues and organ systems, which may reflect the significant cell proliferation and the high mutagenicity of these tissues (5). Primary immunodeficiency diseases (PIDs) are a major category of disorders in which somatic reversion has been frequently described (4). The first case of reversion mosaicism in PIDs was recognized because of a milder than expected clinical phenotype in a patient with adenosine deaminase (ADA) deficiency (6). Reversion mosaicism has subsequently been found in some PIDs such as X-linked severe combined immunodeficiency (X-SCID) (5, 7–13), recombination activating gene 1 (RAG1) deficiency (14, 15), CD3ζ deficiency (16–19), and Wiskott–Aldrich syndrome (WAS) (20–36). The establishment of reversion mosaicism has modified the clinical phenotype of these disorders in which revertant cells have a selective advantage *in vivo* (5). The beneficial consequence of somatic reversion has paved the way to gene therapy, based on gene addition through viral-mediated transfer of the wild-type copy of the gene, which has been applied for curative therapy for ADA-SCID, X-SCID, and WAS (37, 38). Here, we review the newer findings of reversion mosaicism in PIDs and describe clinical implications for understanding this phenomenon.

## PIDs Associated With Reversion Mosaicism

### WAS

WAS is a rare X-linked disorder characterized by thrombocytopenia, eczema, susceptibility to infections, autoimmunity, and lymphoreticular neoplasia. The responsible gene, *WAS*, encodes the WAS protein (WASp). WASp, an essential regulator of actin cytoskeleton remodeling, is only expressed in hematopoietic cells and is involved in multiple functions in immune cells. WASp-deficient T-cells fail to form and stabilize the immunological synapse, thereby resulting in defective polarization of T-cells toward antigen-presenting cells. WASp also has a critical role in T- and B-cell interaction, B-cell homeostasis, phagocytosis, cytolytic function of natural killer (NK) cells, and FAS-mediated apoptosis. Various roles of WASp in innate and acquired immunity have been progressively reported, but the full function of WASp remains to be clarified (39–42).

Somatic reversion seems to occur frequently in WAS patients, as can be expected from the fact that at least 40 cases in 17 studies have been published to date (20–36) (**Table 1**). The European Society for Immunodeficiencies survey of 40 groups/laboratories worldwide documented that WASp reversions occurred in approximately 11% (30/272) of WAS patients (22). WASp-expressing revertant cells have been detected as early as 3 months and as late as the 4th decade of life. Revertant cells have been detected among T-cells, B-cells, and NK cells. However, T-cells are the most common subset of lymphocytes to contain revertant cells with percentages ranging from 5% to 80% (**Table 1**). The fraction of revertant CD4^+^ and CD8^+^ T-cells is diverse among each case. In approximately one-half of the observed cases, more than one lymphoid lineage included revertant cells; however, no cases of revertant cells involving myeloid cells have been reported (4, 22, 39) (**Table 1**). The revertant cells can accumulate over time and are preferentially represented among memory T-cells, regulatory T-cells, and NK cells, which indicates that a reversion mutation can confer significant selective advantage to the revertant cell population when it restores WASp function (27, 29, 30, 33–35). The expansion of revertant cells, which has been observed in *in vitro* culture, also supports this notion (21). An important note is that the appearance of WASp revertant cells in circulation does not necessarily confer a clinical benefit for the WAS phenotype. In many patients, the presence of revertant cells does not prevent the occurrence of life-threatening complications, whereas several patients have presented with a milder clinical course than expected, based on their mutations (40). Clinical outcome after reversion probably depends on at least following factors: the timing of reversion during cell differentiation process, the type and function of cells in which reversion occurs, and the size and diversity of revertant cell population (27). In addition, autoimmune manifestations may be caused by the presence of residual WASp-negative autoreactive lymphocyte *per se*, even if WASp-expressing revertant cells accumulate (27, 28). These findings will offer valuable perspectives on the possible clinical outcomes of gene therapy for WAS patients.

**Table 1:** Somatic revertant cases of WAS.

Number of patients	Type of reversion	Revertant cell	Reference
1	Second-site mutation	Lymphocytes	(20)
1	Back mutation	CD4^+^T, CD8^+^T	(21)
3	A 6-bp deletion (DNA slippage)	CD4^+^T, CD8^+^T	(29, 31)
2	Second-site mutation (19-bp deletion)	CD4^+^T, CD8^+^T, B	(30, 32)
1	Back mutation (1-bp deletion)	NK	(33)
1	Second-site mutation	CD4^+^T, CD8^+^T, NK	(34)
1	Back mutation (1-bp insertion)	T, B, NK	(35)
1	Back mutation	CD4^+^T, CD8^+^T, γδT	(36)
30^a^	Back mutation or second-site mutation	T, B, NK	(22)
2	Multiple second-site mutations	CD4^+^T, CD8^+^T, B, NK	(23)
1	Multiple reversions (back mutation, site-specific substitutions and second-site mutations)	CD4^+^T, CD8^+^T, B	(24, 26)
2	Multiple second-site mutations	CD4^+^T, CD8^+^T, B	(25)
1	Second-site mutation	CD4^+^T, CD8^+^T	(27)
1	Back mutation	CD4^+^T, CD8^+^T, NK	(28)

a Some cases overlap.

WAS, Wiskott–Aldrich syndrome; NK, natural killer.

Based on a molecular point of view, back mutations and second-site mutations are equally common mechanisms that restore WASp expression (4, 39) (**Table 1**). The mutational hotspots for reversion have not been indicated; however, 30% of reversions occur in exon 10, as distinct from the fact that most loss-of-function mutations in *WAS*, primarily missense mutations, are in exons 1 to 4 (22, 43). Of note, more than 30 different genotypic changes have been detected in primary T-cell clones from an adult WAS patient carrying the c.995C>T nonsense mutation in exon 10 (24, 26). However, revertant cells with multiple genotypes within the same individual has been identified in two WAS siblings who carried a c.58C>T nonsense mutation in exon 1 and in other two siblings who carried a c.G1305del mutation, which is predicted to cause a truncated protein that lacked the C-terminal verprolin homology, cofilin homology, and acidic domains (23, 25). This phenomenon also has been identified in other immunological disorders (e.g., CD3ζ deficiency, X-SCID, and RAG1 deficiency) and nonimmunological disorders (e.g., epidermolysis bullosa) (11, 14, 19, 44). Conventional direct sequencing analysis may not allow the detection of multiple changes if they do not represent a significant fraction of existing genotypes. Therefore, a possibility is that the frequency of multiple revertant genotypes in WAS (and possibly other genetic disorders) has been under-reported because of technical reasons. More sensitive and efficient technologies such as deep sequencing technologies and droplet digital polymerase chain reaction (PCR) can be effective for detecting minimal fraction in reversion mosaicism. However, the fact that the clinical history of WAS patients often allows long-term management in the absence of resolutive treatment may allow sufficient time for revertant cells of diverse genotypes to individually accumulate (26, 40).

The reason for the high incidence of reversion mosaicism in WAS, compared to other PIDs, is unclear. The possible mutagenic effect of chronic antibiotic exposure or the high proliferation rate in lymphoid cells and consequently increased DNA polymerase mistakes, which are derived from inadequate immune response of WAS patients to infectious agents, may have a causative role in mutagenicity in WAS patients (40). However, no known involvement of WASp in DNA replication, proofreading, and DNA repair exists, and the general mutation rate does not appear to be increased in WAS patients (26). In addition to cytoplasmic functions, several nuclear functions of WASp such as gene transcription and the maintenance of genomic stability have recently been elucidated (45–52). Recent research has demonstrated that nuclear WASp is critical in preventing the accumulation of a genome-destabilizing nucleic acid structure in human T-cells that is called the “R-loop” (i.e., a three-stranded nucleic acid structure consisting of an RNA : DNA duplex and a displaced nontemplate single strand DNA) (51). It also has an important role in correcting existing double strand breaks in human B-cells (52). Moreover, another study revealed a novel function of WASp in the DNA-damage-induced Golgi dispersal response, and its disruption as a contributor to radiosensitivity in T- and B-cells (53). WASp accordingly has critical functions in maintaining a stable genome in human T- and B-cells. The precise mechanism in the high frequency of reversion mosaicism in WAS patients remains unclear; however, WASp-deficiency-induced genomic instability may have an important role in the high incidence of somatic reversions in WAS patients.

### ADA Deficiency

ADA deficiency is an autosomal recessive disorder that represents a SCID phenotype caused by the excessive accumulation of toxic purine nucleoside metabolites. Enzyme replacement therapy (ERT) with polyethylene glycol-ADA eliminates toxic metabolites and protects lymphocytes, thereby restoring the immune function. ERT is efficient for temporal adjunct therapy before hematopoietic stem cell transplantation (HSCT) or gene therapy (6, 54–58). In the middle of 1990s, Hirschhorn et al. reported two unrelated ADA-SCID patients who presented with progressive clinical improvement and biochemical and immunological remission (6, 54). They believe that the patients’ clinical course could have been modified by somatic reversion in which revertant cells had a selective advantage; however, because of the absence of parental genetic analysis, a cause of the genetic mosaicism in one of the patients could not be distinguished from a postzygotic somatic mutation (6, 54).

ERT has been used because it causes a rapid improvement in immune reconstitution, even in somatic mosaicisms caused by reversion mutations. In three of four revertant patients previously reported who underwent ERT, wild-type or second-site revertant cells decreased markedly during ERT, although its biochemical and immunological effects and clinical outcomes differed among them (55–58). This finding highlights the possibility that ERT reduces the selective advantage of revertant cells. However, immune reconstitution and clinical improvement in these patients could depend on several factors such as the lineage of cells in which reversion occurred and the duration of ADA exposure. These findings provide insights into the immunological and clinical effects of gene therapy, especially in the management of ADA patients by combining gene therapy with ERT, with regard to the appropriate timing and duration of ERT.

Gene therapy is a therapeutic strategy for ADA-SCID patients and increasing evidence suggests that T memory stem cells (T_SCM_) are expected to be a potential target because of its long-persisting memory and stem-cell nature (59). Notably, recent study with ADA-SCID patients uncovered that gene-corrected T_SCM_ can persist and preserve functional T-cell pool *in vivo* for up to 12 years without oncogenic feature (59). This finding indicates that T_SCM_ gene correction is a crucial strategy for the T-cell-based gene therapy.

### X-SCID

X-SCID is the most frequent form of SCID, which is caused by mutations in the gene encoding the common gamma chain (γc) of the interleukin-2 receptor (*IL2RG*). In the absence of a functional γc, early lymphoid progenitor cells are unable to achieve the normal development of T-cells and NK cells. Most patients who present with SCID in infancy have a poor survival beyond 2 years without immune reconstitution therapy. However, hypomorphic mutations or reversion mutations of the *IL2RG* gene could result in a mild phenotype (i.e., late-onset combined immunodeficiency). In previous reports, five of seven revertant patients actually recovered γc expression and presented with the mild phenotype (5, 7–13) (**Table 2**). Some of these patients exhibited polyclonal expansion of the T-cell receptor variable β (TCR Vβ) repertoire and a restored response to mitogens (5, 11). In these cases, reversion may occur in progenitor T-cells before the stage in which T-cells undergo TCR rearrangement. In addition, in recently reported cases, reversion was detected in NK cells or B-cells and T-cells, thereby indicating that reversion may occur at the level of the T/NK progenitor or common lymphoid progenitor (12, 13). By contrast, in another report, a patient had multiple reversions with a different proportion in each cell lineage (11) (**Table 2**). This observation suggests that reversion may occur more frequently than previously believed and may have the potential to ameliorate immunological and clinical presentations of X-SCID.

**Table 2:** Clinical and genetic features of revertant cases of X-SCID.

Germline mutation	Type of reversion	Revertant cell	Clinical impact	Reference
c.343T>C	Back mutation	CD4^+^T, CD8^+^T	Patient presented with a mild phenotype, but subsequentially underwent HSCT because of recurrent infections	(7)
IVS1+5G>A	Second-site mutation	T (only skin infiltrated)	Omenn syndrome	(9)
c.466T>C	Back mutation	αβT, γδT	Mild phenotype	(10)
c.284-15A>G	Multiple reversions	CD4^+^T, CD8^+^T	Mild phenotype	(11)
c.655T>A	Back mutation	CD4^+^T, CD8^+^T, γδT	Mild phenotype	(5)
c.260T>C	Back mutation	CD4^+^T, CD8^+^T, B	Mild phenotype	(12)
c.172C>A	Back mutation	CD8^+^T, NK	Patient died of graft failure and fungal infection after HSCT	(13)

X-SCID, X-linked severe combined immunodeficiency; HSCT, hematopoietic stem cell transplantation; NK, natural killer.

By contrast, an adverse clinical effect of reversion was raised by the observation of an X-SCID patient who presented with an Omenn syndrome (OS)-like phenotype (9) (**Table 2**). In this patient, revertant T-cells were only detected in skin infiltrates, not in peripheral blood, which indicated that the clonal expansion of revertant cells in response to local factors such as infections or autoantigens may be implicated in such a distinctive phenotype. This case indicates that somatic reversion is a possible cause of clinical improvement and for diverse and complicated presentations.

### RAG1 Deficiency

RAG1 is a component of the RAG complex promoting V(D)J recombination in precursor lymphocytes by which highly diverse immunoglobulins and TCR genes are generated. Severe/null defects in the *RAG1* gene cause T^-^B^-^SCID, whereas leaky mutations and somatic reversions are responsible for the development of OS (14, 15, 60, 61). In a previous report of a patient with RAG1 deficiency who presented with the OS phenotype, multiple second-site mutations that generated a partially functional RAG1 molecule could have contributed to insufficient immunological reconstitution (14). In another report, a true back mutation, which was believed to occur in a limited pool of progenitor T-cells, may have been involved in the presentation of the OS phenotype in a patient (15). Both of these patients had a restricted pattern in the TCR Vβ repertoire and activated T-cell markers. In the first patient, not all mutations were commonly shared by CD4^+^ and CD8^+^ T-cells (14). However, a complete defect in RAG activity results in differentiation arrest before the stage of double-negative to double-positive transition (62). These findings suggest that other precedent mutations may be followed by these second-site mutations, which occur after CD4/CD8 lineage commitment.

### CD3ζ Deficiency

CD3ζ, also called as CD247, is a subunit of the TCR complex that is required for its assembly and for surface expression, which is important for TCR-mediated signal transduction. TCR complexes lacking the CD3ζ chain cumulate in the Golgi apparatus instead of moving onto the plasma membrane and are shunted to lysosomes for degradation. The CD3ζ chain contributes to peripheral T-cell activation and intrathymic T-cell differentiation. The lack of CD3ζ expression results in a severe but incomplete block of T-cell differentiation at the double-positive stage (16, 17, 63).

Three patients with CD3ζ deficiency were previously found to have somatic reversion mutations in *CD3Z* gene (16–19). The first patient, who carried a germline mutation within the intracellular first immunoreceptor tyrosine-based activation motif domain and presented with the SCID phenotype, harbored three second-site mutations that partially rescued membranous TCR expression but functioned poorly (16). Ten percent of T-cells were revertant T-cells expressing normal levels of the TCR-CD3 complex and were polyclonal. All of the cells were CD4^+^ T-cells carrying one of three reversion mutations. Each of the three mutations was found in populations harboring different rearrangements of TCR Vβ genes. This finding suggests that the reversions may occur before the V(D)J recombination at the double-negative stage. The lack of revertant cells in CD8^+^ T-cells indicates that these three mutations may probably have little selective advantage for CD8^+^ T-cells. The second revertant case involved a germline mutation within the initiation codon, which inhibited translation. This patient harbored revertant cells with a true back mutation or a compensatory second-site mutation, which caused restoration or substitution of the initiation codon; however, the frequency of revertant cells in T-cell compartment was very low (17). Revertant T-cells are capable of expanding in response to TCR stimuli *in vitro* but may not be sufficient to repopulate the T-cell compartment and achieve immunological reconstitution *in vivo*. The *CD3Z* gene may have intrinsic mutability so as to result in multiple reversions, as reported in two patients (18). This finding has been substantiated by the latest report of a revertant patient with multiple second-site mutations who presented with the mild phenotype (19). This patient carried a homozygous 2-bp deletion mutation (c.43_44delCA) within *N*-terminal signal peptide in the *CD3Z* gene. Deep sequencing analysis revealed 52 somatic variants of which 49 variants restored the reading frame, most of which retained the characteristic amino acid distribution functioning as signal peptides but were not fixed to the primary sequence. A surprising finding is that 1 year after the first analysis, 23 somatic variants, which included nine novel variants, were detected with a different proportion from that of the first analysis, which may reflect the varying antigen stimulations. The multiple variation in the amino acid sequence caused by reversion mutations may reflect that signal peptides permit the fluctuation of the amino acid sequence, which may be distinct from the integral sequence for protein function. This case demonstrates that the location of the germline mutation can affect the spectrum of the revertant pool.

### Other PIDs Presenting With the SCID Phenotype

The first case of revertant mosaicism in Janus kinase 3 (JAK3) deficiency was reported in a consanguineous family with two affected siblings (64). They presented with a relatively mild phenotype with CD4 lymphopenia, which manifested as combined immunodeficiency. A novel homozygous missense mutation (c.3196T>C) was identified in both patients. One of these patients presented with somatic mosaicism by a back mutation in CD8^+^ T-cells; in the other patient, the same back mutation was detected in CD4^+^ and CD8^+^ T-cells. The patient with CD4^+^ and CD8^+^ revertant T-cells presented with a milder phenotype than her counterpart. Therefore, somatic reversion in CD4^+^ T-cells may have contributed to the attenuation of disease severity. However, JAK3-signaling analysis showed that the presence of revertant cells had no effect on the residual JAK3-dependent signaling. Hence, the hypomorphic nature of this mutation rather than reversion mutation may have been associated with the milder phenotype in the second patient.

In a study of Chinese patients with DNA ligase IV (LIG4) deficiency, one of seven patients presented with reversion mosaicism (65). The germline genotype of this patient was a compound heterozygote (c.833G>T; c.935delC) in the *LIG4* gene; however, wild-type clones and simultaneously generated clones, which contained both inherited mutations, were detected with TA cloning analysis. This finding indicates that intragenic recombination was a probable mechanism of somatic repair of *LIG4* mutations. The reversion event may occur at an early stage of embryonic development because these clones were obtained from T-cells, NK cells, granulocytes, and oral mucosa cells in different proportions. However, the somatic reversion was insufficient to reconstitute clinical and immunological phenotype in this patient.

A patient with interleukin (IL)-7 receptor α deficiency, who had compound heterozygous mutations (one single nucleotide variant and one intragenic copy number variant involving one exon), presented with an atypical clinical course and late onset (66). Mosaicism for the wild-type allele in the single nucleotide variant position was unexpectedly identified in whole exome sequencing. However, whether it originated from somatic reversion, maternal engraftment, or both was unproven.

### XL-EDA-ID (NEMO Deficiency)

X-linked anhidrotic ectodermal dysplasia with immunodeficiency (XL-EDA-ID) is caused by hypomorphic mutations in *IKBKG* gene, which encodes nuclear factor-κB (NF-κB) essential modulator (NEMO). NEMO has an important role in activating inhibitor of NF-κB (IκB) kinase, which phosphorylates and degrades IκB to activate NF-κB. A defect in NEMO causes various abnormalities in the signal transduction pathway involving NF-κB, including the IL-1 family protein receptors, the Toll-like receptors, CD40, and the tumor necrosis factor (TNF) receptor (67, 68). Patients with XL-EDA-ID present with various immunological phenotypes such as reduced production of proinflammatory cytokines in response to lipopolysaccharide and IL-1 family protein stimulation, dysregulated immunoglobulin synthesis, defective anti-polysaccharide antibody synthesis, and NK cell dysfunction (67, 68).

In a Japanese nationwide survey, as many as 90% (9/10) of XL-EDA-ID patients presented with somatic reversion mosaicism (69). In these patients, most revertant cells were detected in CD4^+^ and/or CD8^+^ T-cells, a low number of revertant cells was detected in B-cells, and no revertant cells were detected in monocytes. One sibling who harbored a duplication mutation in the *IKBKG* gene also showed reversion mutation in approximately 50% of CD56^+^ NK cells (70). In two siblings, the revertant CD8^+^ T-cells primarily presented with the memory/effector phenotype and the restricted pattern of the TCR Vβ repertoire (69, 70). The high incidence of somatic mosaicism may reflect a strong selective advantage for NEMO-expressing revertant cells *in vivo*, especially in the T-cell lineage. However, the precise role for NEMO in the development and homeostasis of each cell lineage has not been fully elucidated. The clinical impacts of somatic mosaicism in XL-EDA-ID patients were not demonstrated in the studies (69, 70).

By contrast, the clinical impact of NEMO reversion has been demonstrated in another revertant patient who presented with refractory inflammatory colitis (71). The mechanism underlying the NEMO colitis was demonstrated in a mouse model of intestinal epithelium-specific NEMO deficiency in which intestinal epithelial cells exhibited increased sensitivity to TNFα-induced apoptosis and caused disruption of the epithelial barrier, resulting in chronic intestinal inflammation (72). In this patient, the NEMO-deficient intestinal epithelium might be further damaged by TNFα-producing mononuclear cells because of the reversion mutation of NEMO. The patient’s peripheral TNFα-producing cells reduced with repeated anti-TNFα antibody administrations, thereby causing the clinical improvement. Thus, NEMO reversion may have a deleterious role in the intestinal inflammation.

Owing to the presence of a pseudogene (i.e., *IKBKGP1*), the genetic diagnosis of XL-EDA-ID is difficult to determine when using only genomic DNA sequencing analysis. It should be confirmed by sequencing analysis of NEMO cDNA or long-range PCR amplicon using primers that do not amplify the pseudogene. The presence of somatic mosaicism can cause a misdiagnosis of XL-EDA-ID when a normal revertant cDNA sequence can be selectively amplified with PCR or when most of each cell lineage consists of NEMO-expressing revertant cells (69).

### LAD-1

Leukocyte adhesion deficiency type 1 (LAD-1) is caused by a genetic defect in the *ITGB2* gene, which encodes the common chain of the β2 integrin family (CD18). The adhesion of leukocytes to the endothelium is primarily defected, thereby causing abnormal leukocyte extravasation. Patients are usually affected with recurrent bacterial infections and impaired wound healing without pus formation. The severity of the clinical phenotype is directly associated with the degree of CD18 deficiency (73). To date, five revertant patients harboring CD18-expressing cells have been reported in LAD-1 (74–76). The first patient had a compound heterozygous mutation and carried a reversion to the normal sequence in one of the disease-causing mutations only within a small fraction of CD8^+^ T-cells. This fraction was monoclonal, which indicated that reversion may occur in a committed hematopoietic lineage and eventually gain a selective advantage (74). In three additional patients, all of whom presented with gastrointestinal manifestations, 5% to 20% of revertant cells were detected among CD8^+^ T-cells and exhibited a restricted pattern of the TCR Vβ repertoire (75). In all four cases, revertant CD8^+^ T-cells represented the memory/effector phenotype. In addition, a functional study revealed that reversion mutations showed the recovery of superantigen-induced proliferation and adhesion to specific ligands *in vitro* (75). Taken together, these findings suggest that reversion mutations may exhibit functional recovery and a proliferative advantage and lead to the acquisition of the immunological memory in response to antigen stimulation, especially in CD8^+^ T-cells.

In these previous patients, susceptibility to infections was not ameliorated, probably because of the lack of reversion in granulocytes. By contrast, as with the three revertant patients with gastrointestinal manifestations, the latest case of LAD-1 in a patient, who was presumably a revertant patient because of the presence of minimal CD18^+^ fraction in circulating CD8^+^ T-cells, was also affected with severe colitis, which was endoscopically compatible with Crohn’s disease (76). The inflammatory response at barrier sites such as the oral mucosa and probably in the skin and gastrointestinal tract in LAD-1 may be caused by infections because of relative tissue neutropenia and by a defect in the phagocytosis of apoptotic neutrophils by tissue macrophages (i.e., efferocytosis), which acts as signal to down-regulate IL-23 and IL-17 responses (77). However, the clinical impact of the presence of CD18-expressing CD8+ T-cells regarding the gastrointestinal inflammatory response remains to be clarified.

### XLP-1

X-linked lymphoproliferative disease type 1 (XLP-1) is caused by loss-of-function mutations in *SH2D1A*, which encodes SLAM-associated protein (SAP). Patients with XLP-1 are highly susceptible to Epstein–Barr virus (EBV) infection because of the impaired activation and cytotoxicity of CD8^+^ T-cells. They also develop hypogammaglobulinemia because of impaired CD4^+^ T-cell function (78, 79). In the first study of somatic reversion in XLP-1, eight patients represented a small fraction of SAP^+^ revertant cells in CD8^+^ T-cells, except for one patient who also presented with SAP^+^ fraction in NK cells (78). Most revertant CD8^+^ T-cells lay in the CD45RA^-^CCR7^-^ effector memory T-cell compartment and maintained a stable number over decades. In addition, SAP^+^ revertant CD8^+^ T-cells showed more proliferation than did the SAP-deficient counterpart and exhibited functional recovery in response to EBV-specific stimulus. As an alternative, in the latest Japanese nationwide survey of XLP-1, three of 18 patients, who were alive at the time of this study, had a longer survival without HSCT, despite having a history of EBV infection (79). A remarkable finding is that all three patients had 3% to 7% of the SAP^+^ fraction in CD8^+^ T-cells, and one patient had a smaller fraction of SAP^+^ cells in CD4^+^ T-cells. SAP^+^ CD8^+^ T-cells mostly lay in the effector memory T-cell population and showed proliferation and functional recovery in response to EBV-specific stimulus, compatible to those of healthy controls. In addition, in the patient who harbored revertant SAP^+^ cells in the CD4^+^ T-cell population, intracellular IL-10 expression and inducible costimulatory expression were predominantly observed in the revertant SAP^+^ CD4^+^ T-cells. This finding suggested that the reversion event conferred partial reconstitution of humoral immunity in this patient. The findings in these studies collectively suggested that a reversion mutation provides the ability for proliferation and the acquisition of effector function for revertant CD8^+^ T-cells in response to B-cells (i.e., the reservoir of EBV) and eventually leads to expansion of revertant T-cells in correlation with EBV infection. The fact that a relatively small fraction of revertant cells modified the fatal clinical phenotype of XLP-1 suggested that gene therapy may potentially be an effective strategy for XLP-1.

### DOCK8 Deficiency

Dedicator of cytokinesis 8 (DOCK8) is a guanin nucleotide exchange factor for the Rho-GTPase CDC42, turning it into the active, GTP-bound form. GTPase activation induces dynamic actin cytoskeleton rearrangement, leading to immunological synapse formation, migration, adhesion, and cytolytic granule release. DOCK8 also exhibits an actin-independent function, including the regulation of STAT3 phosphorylation and nuclear translocation. Hence, DOCK8 deficiency impacts innate and adaptive immune responses (80).

Of note, somatic reversions occur in 13% to 50% of patients with DOCK8 deficiency (81, 82). The high frequency of somatic reversions may reflect the *DOCK8* gene’s location within a recombination hotspot that is characterized by many subtelomeric repetitive sequences (81). Reversion is detected more frequently in T-cells, especially in a high proportion of CD8^+^ T-cells, rather than in NK or B-cells, but it is not detected in monocytes (81, 82). Revertant T- and B-cells exhibit expansion *in vitro* and *in vivo* and restore CD8^+^ T-cell cytotoxicity, CD4^+^ T-cell cytokine production, and memory B-cell generation (82). These findings indicate that DOCK8 somewhat exerts a proliferative and survival advantage in numerous lymphoid lineages and has a key role in many fundamental aspects of lymphocyte biology. DOCK8-expressing cells are predominantly enriched in the memory compartment of CD4^+^ T-cells and B-cells rather than in the corresponding naïve compartment. By contrast, the difference in the proportions of DOCK8-expressing cells in the CD8^+^ T-cell population was not as significant as in CD4^+^ T-cells and B-cells (81, 82). This finding reflects a possible differential role of DOCK8 among each lymphoid lineage and in CD4^+^ and CD8^+^ T-cell differentiation. Based on a clinical perspective, the increase in revertant cells can delay the progression of the disease, but do not necessarily reconstitute the whole clinical phenotype and do not abrogate the need for HSCT (81, 83–85). By contrast, the three patients reported by Pillay *et al.* exhibited restored protein expression and spontaneous improvement in the clinical phenotype (82). These findings from these patients provide evidence that gene therapy is a promising prospect for treating DOCK8 deficiency.

### CARD11 Deficiency

In 2015, the first case of somatic reversion in caspase recruitment domain-containing protein 11 (CARD11) deficiency was reported (86). The patient was one of two Turkish siblings who were born to consanguineous parents. She had postnatally acquired cytomegalovirus infection and presented with the OS-like phenotype, which included erythroderma and lymphadenopathy. She carried a homozygous germline nonsense mutation (c.450C>A) in the coiled-coil domain in the *CARD11* gene, which impaired NF-kB signaling and IL-2 production. A somatic second-site mutation (c.449G>T) was detected in the same codon, which restored protein expression, thereby leading to a partial functional restoration in a subset of T-cells. The revertant cells were mostly in tissue-infiltrating CD4^+^ and CD8^+^ T-cells, but not in granulocytes and fibroblasts, with a highly restricted T-cell repertoire. This finding indicated that reversion may occur before CD4/CD8 lineage commitment in progenitor T-cells. As demonstrated in mouse studies, CARD11 is also essential for the thymic development of FoxP3^+^ regulatory T-cells, which significantly contributes to peripheral tolerance of T-cells (87, 88). These siblings indeed had a lack of regulatory T-cells in the peripheral blood or in lymph node biopsy. Along with the selective advantage of virus-specific revertant T-cells in response to the persistent stimuli by chronic cytomegalovirus infection, the lack of regulatory T-cells may also have a causative role in the development of the OS-like features.

### ARPC1B Deficiency

Actin-related protein 2/3 complex subunit 1B (ARPC1B) is a component of the actin-related protein 2/3 complex, which interacts with WASp to induce actin polymerization and generate new branched actin filament networks in the context of cell migration, endocytosis, vesicular trafficking, and cytokinesis (89). ARPC1B exerts a regulatory role for the assembly and maintenance of the actin-related protein 2/3 complex, and its disruption results in morphological, functional, numerical aberrations in platelets, defect in neutrophil motility and chemotaxis, and functional deficiency in T-cells and NK cells (89–93). In 2017, somatic reversion in ARPC1B deficiency was first reported in two patients from unrelated families (89). In these patients, somatic reversion restored ARPC1B protein expression and was associated with a restricted TCR repertoire. One patient harbored revertant cells within the CD8^+^ T-cell compartment, but not in CD4^+^ T-cells and B-cells. The other patient harbored revertant cells in the CD8^+^ T-cell and NK cell compartments. In addition, ARPC1B^+^ revertant CD8^+^ T-cells displayed an improvement in T-cell migration. These findings suggested that reversion mutations in *ARPC1B* provide a preferential advantage in CD8^+^ cytotoxic T-cells and NK cells, which is consistent with the fact that the absence of ARPC1B disrupts the proliferation capacity of cytotoxic T-cells (93). Furthermore, revertant CD8^+^ T-cells were only enriched in effector memory T-cells, T effector memory-RA^+^ cells, and T_SCM_, but not in naïve and central memory T-cells. This finding indicated that part of the selective pressure may have occurred on antigen stimulations, thereby leading to the acquisition of the immunological memory. Neither patient in the study seemed to have an improvement in their clinical phenotype and one of the patients underwent HSCT due to recurrent infections and refractory autoimmune/autoinflammatory manifestations. The restoration of ARPC1B expression may partially reconstitute T-cell function and proliferation; however, more observations are required to assess whether genetic reconstitution provides the clinical improvement in ARPC1B deficiency.

### MYSM1 Deficiency

Myb-like, SWIRM, and MPN domains 1 (MYSM1) is a histone deubiquitinase that specifically deubiquitinates the K119-monoubiquitinated form of histone 2A, a chromatin marker of gene transcription silencing. MYSM1 reverses the transcription repression of genes that are involved in hematopoietic stem cell homeostasis, hematopoiesis, and lymphocyte differentiation (94). Spontaneous *in vivo* reversion to normal was recently reported in a male patient with MYSM1 deficiency who harbored a missense mutation (c.1967A>G) that affected the stability of the protein and damaged histone deubiquitinase activity (94). He presented with a complete lack of B-cells, T-cell lymphopenia, bone marrow failure, and developmental abnormalities. During the course of his treatment, he showed spontaneous improvement in the number of lymphoid and myeloid lineages. Analysis of bone marrow mononuclear cells revealed the complete restoration of B-cell development. Circulating B-cells exhibited polyclonal pattern of B-cell receptor IgM and IgG repertoire. A surprising finding was that a genetic reversion to normal was detected in virtually all hematopoietic stem cells (HSCs), and subsequently detected in approximately 100% of circulating B-cells, NK cells, and monocytes, and in 63.5% of circulating T-cells. These findings suggested that the reversion event may have provided a selective advantage for these cells *in vivo* and completely corrected immunological abnormalities and bone marrow failure in this patient. This observation suggests that clinical application of gene therapy will be effective for immunodeficiency and bone marrow failure in patients with MYSM1 deficiency.

### WHIM Syndrome

Warts, hypogammaglobulinemia, infections, and myelokathexis (WHIM) syndrome is usually caused by a gain-of-function mutation in the *CXCR4* gene, which is in chromosome 2. CXCR4 signaling is a suppressive modulator of HSC and neutrophil migration from bone marrow. Its gain-of-function mutation results in myelokathexis, which is a characteristic of this disorder. In addition, the *CXCR4* gain-of-function mutation may cause a multisystem and combined immunodeficiency disease because of its broad expression in hematopoietic and nonhematopoietic cell types (95). A female patient with WHIM syndrome, designated as WHIM-09, experienced a spontaneous phenotypical remission (96). In this patient, chromothripsis, a complex genetic process characterized by scattering, rearrangement, inversion and deletion of genomic element on one or a few chromosomes (97), affected one copy of chromosome 2 and deleted 164 genes, including the mutated copy of *CXCR4*, probably in a single HSC from the patient. This event conferred to the modified HSC a strong selective advantage over germline mutated cells and corrected the defects in the myeloid and erythroid lineage. By contrast, the lymphoid lineage and epithelial cells were not affected by the reversion event. Therefore, the patient had a somatic mosaic of WHIM cells and reverted non-WHIM cells. The patient has been healthy for at least 20 years since the estimated time point of the reversion event. These findings suggest that the adaptation of genome editing technology to inactivate the mutant *CXCR4* allele in autologous HSC is a potential curative strategy for WHIM syndrome.

### GATA2 Deficiency

GATA-binding protein-2 (GATA2) is a transcription factor that is involved in the development of HSCs and is essential for definitive hematopoiesis. Heterozygous mutations in *GATA2* results in GATA2 haploinsufficiency and causes qualitative impairment in HSC function. GATA2 deficiency is characterized by various hematopoietic and nonhematopoietic features such as multilineage cytopenias (especially monocytopenia and B-cell and NK cell lymphopenia), hematologic malignancies, immunodeficiency, pulmonary alveolar proteinosis, lymphedema, and sensorineural hearing loss (98, 99). The first somatic revertant case of GATA2 deficiency was revealed by the diagnosis of a man’s two affected sons who presented with the typical phenotype (98). They harbored a heterozygous mutation in *GATA2* (c.216C>A; p.Y72*), which caused a premature stop codon and loss of the two DNA-binding zinc fingers and the nuclear localized signal. By contrast, their father was asymptomatic and displayed normal immunophenotyping. A surprising finding was that he harbored the pathogenic mutation, which was identical with that of his sons, in sperm and skin fibroblasts. However, 93% of leukocytes carried the silent somatic mutation (c.216C>T; p.Y72Y), which indicated somatic reversion. Sorted monocytes, T-cells, B-cells, and NK cells carried this silent somatic variant in heterozygosity, which indicated that somatic reversion may occur at the HSC stage. The patient remained asymptomatic with no hematopoietic or nonhematopoietic manifestations at the point of time in this study when he was 61 years old, despite the fact that he did not carry the silent somatic variant in the nonhematopoietic lineage. This finding indicates that the restoration of hematopoietic cells is sufficient for preventing the occurrence of hematopoietic and nonhematopoietic manifestations in GATA2 deficiency. These observations suggest the potential advantage of gene therapy. However, more observations are necessary for understanding the full details of somatic reversion in GATA2 deficiency.

### SAMD9/SAMD9L Syndrome

Germline mutations in sterile alpha motif domain protein 9 (*SAMD9*) and its paralogue SAMD9-like (*SAMD9L*), which are located in tandem on chromosome 7q21, are associated with human syndrome with a propensity for bone marrow failure and myelodysplastic syndrome (MDS) with monosomy 7 and 7q deletion (100, 101). *SAMD9* and *SAMD9L* gain-of-function mutations represent a strong growth-suppressive effect because of the defective endosomal turnover of cytokine receptors such as the epidermal growth factor receptor (100). Somatic reversion events, which remove the mutant *SAMD9* and *SAMD9L* allele, occur *via* monosomy 7, chromosome arm 7q deletion, second-site loss-of-function mutation, and CN-LOH (102–110). Cells that lose the mutant allele may gain a proliferative advantage, relative to the growth restriction imposed in mutation-carrying cells, and thereby result in reversion mosaicism. Any somatic reversion event may be temporarily beneficial for hematopoiesis; however, the loss of the germline mutated copy *via* monosomy 7 and 7q deletion results in haploinsufficiency (3, 100, 108). In fact, in an original case series of myelodysplasia, infection, restriction of growth, adrenal hypoplasia, genital phenotypes, enteropathy syndrome, which was initially recognized as a disease caused by *SAMD9* gain-of-function mutations, two of 11 patients developed myelodysplastic syndrome (MDS) with monosomy 7 (102). Several additional cases have subsequently been reported and affected patients have a propensity to develop MDS/acute myeloid leukemia with monosomy 7 and 7q deletion (102, 106, 107, 109). Like patients with *SAMD9* mutations, patients with *SAMD9L* gain-of-function mutations are also predisposed to the development of MDS/acute myeloid leukemia with monosomy 7 and 7q deletion (108, 109). CN-LOH and second-site loss-of-function mutation can apparently rescue the hematopoietic defect without MDS/acute myeloid leukemia predisposition in both disorders (100, 103–105, 107–110).

In the recent investigation of pediatric MDS cohort, 8% of the consecutively diagnosed patients harbored germline *SAMD9*/*SAMD9L* mutations (101). Of the patients with *SAMD9*/*SAMD9L* mutations, 61% underwent somatic reversion, of whom 51% had benign (CN-LOH or second-site mutation) and 95% had maladaptive nature (monosomy 7 and 7q deletion). Furthermore, bone marrow single-cell sequencing revealed multiple competing reversions in individual patients. As shown in the study, somatic reversion in SAMD9/SAMD9L syndrome is highly prevalent and diverse, and changes in clonal diversity may modify clinical outcomes of the patients.

## Discussion

Over the past decades, observations of somatic reversion in PIDs have provided significant insights into the function of genes in different cell types, the frequency and mechanism of genetic changes, and clinical consequences because of the presence of revertant cells. These observations have yielded the notion that reversion events provide a wide-spectrum of molecular and clinical effects in PID patients. In fact, the molecular mechanisms that restore gene expression and function vary from single nucleotide substitutions to large deletions or insertions. Moreover, chromosomal structural change, as seen in WHIM syndrome and SAMD9/SAMD9L syndrome, contributes to complete gene abrogation in PIDs caused by gain-of-function mutations (96, 102–110).

The increased number of reversions in PIDs suggests that reversion mutations may occur much more frequently than previously believed rather than being a significantly rare event that occurs in distinctive situations. The relatively high occurrence of reversion is substantiated by the fact that independent reversions can occur in respective siblings (30, 64, 79) and in respective cell lineages (81). In addition, reversion mutations have been also discovered in myeloid and erythroid lineages and in nonhematopoietic cells, whereas lymphoid lineages are most frequently affected (65, 94, 96, 98, 105, 106) (**Table 3**). Together with these observations, reversion mutations can occur frequently in various cell types and differentiation stages and can be detected when affected cells acquire a proliferative advantage, which can be determined by, at least, the following factors: the type of cell, the timing of reversion, the lifespan of revertant cells, the nature of mutant genes, and extrinsic factors such as persistent antigen stimulations (3, 5, 78). Based on this perspective, even when various cell lineages share the same reversion, identical reversions may occur independently at more differentiated stages rather than from a single less-differentiated common progenitor.

**Table 3:** Other PIDs in which somatic reversion has been detected.

Disease	Type of reversion	Revertant cell	Reference
ADA deficiency	Back mutation	CD4^+^T, CD8^+^T, B, NK	(6, 54–58)
	Second-site mutation		
RAG1 deficiency	Back mutation	CD4^+^T, CD8^+^T	(14, 15)
	Second-site mutation		
CD3ζ deficiency	Back mutation	CD4^+^T, CD8^+^T, NK	(16–19)
	Second-site mutation		
XL-EDA-ID	Loss of the duplicated region	CD4^+^T, CD8^+^T, B, NK	(69–71)
	Back mutation		
LAD-1	Back mutation	CD8^+^T, NK	(74–76)
	Site-specific substitution		
	Second-site mutation		
XLP-1	Back mutation	CD4^+^T, CD8^+^T, NK	(78, 79)
	Site-specific substitution		
DOCK8 deficiency	Back mutation	CD4^+^T, CD8^+^T, B, NK	(81–85)
	Second-site mutation		
	CN-LOH		
	Intragenic recombination		
	Loss of the duplication/deletion mutation		
JAK3 deficiency	Back mutation	CD4^+^T, CD8^+^T	(64)
DNA ligase IV deficiency	Intragenic recombination	T, NK, granulocytes, oral mucosa	(65)
CARD11 deficiency	Second-site mutation	CD4^+^T, CD8^+^T	(86)
ARPC1B deficiency	Back mutation	CD8^+^T, NK	(89)
MYSM1 deficiency	Back mutation	T, B, NK, monocytes	(94)
WHIM syndrome	Chromothripsis	myeloid and erythroid lineage	(96)
GATA2 deficiency	Site-specific substitution	T, B, NK, monocytes	(98)
SAMD9/SAMD9L syndrome	Monosomy 7	BM and PB cells (including myeloid and lymphoid lineage)	(102–110)
	Deletion of 7q		
	Second-site mutation		
	CN-LOH		

PID, primary immunodeficiency disease; NK, natural killer; CN-LOH, copy-neutral loss of heterozygosity; BM, bone marrow; PB, peripheral blood.

Reversion mutations frequently occur in some PIDs, although susceptible genes and locations may not be random. For example, distinctive sequences in *WAS* have been implicated in reversion mutation by means of DNA slippage mechanism or hairpin loop formation (29–32). In addition, the homologous sequence of *IKBKG* (i.e., pseudogene) and subtelomeric repetitive sequences in the *DOCK8* locus may have contributed to the recombination-mediated somatic repair (70, 81, 82). (**Table 3**). Loss-of-function mutations in genes that are involved in genomic stability such as *BLM*, *LIG4*, *FANCA*, *FANCC*, *FANCD2*, and presumably *WAS* may be associated with a high incidence of reversion mutations (3, 51–53). Gene variation analysis, used to evaluate the mutation frequency of PID genes in which reversion events have or have not been described, has revealed that genetic variation is significantly greater for revertant genes than for nonrevertant or control genes, especially in coding sequences (18). The study also demonstrated that the presence of CpG islands was more frequent in revertant PID genes than in nonrevertant genes; however, other factors such as local chromatin structure and accessibility for DNA repair may influence mutation frequency (18). These intrinsic gene properties in collaborating with long-term management using mutagenic agents and prompt cell turnover due to chronic infections and inflammation (which may induce DNA polymerase mistakes) may be involved in the high incidence of reversion mutations.

**Figure 2:**
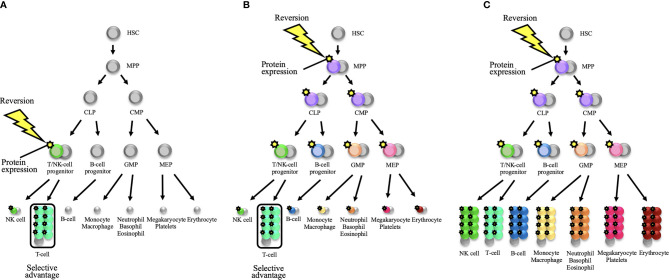
Schematic representations of somatic reversion in the hematopoietic system that eventually results in distinct hematological and immunological reconstitution. **(A)** A somatic reversion event can occur at a late stage of the hematopoietic hierarchy and provide a selective advantage to a restricted lineage. The diagram shows the occurrence of reversion in a T/NK-cell progenitor. When T-cell lineage selectively exhibits a proliferative advantage, somatic mosaicism will be detected only in the T-cell population. This model is applicable to the reversion mosaicism in the context of severe combined immunodeficiency or a T-cell deficiency. **(B)** A somatic reversion event can occur in less-differentiated hematopoietic progenitor cells. For example, when reversion mutation occurs in a multipotent progenitor (MPP), cells from all hematopoietic lineages can theoretically harbor the same reversion mutation. However, when the T-cell lineage selectively represents a proliferative advantage, somatic mosaicism will seemingly be detected only in the T-cell lineage. This selective advantage may depend on intrinsic gene function for T-cell proliferation and/or persistent antigen-specific responses. In disorders with T-cell lymphopenia such as severe combined immunodeficiency, homeostatic expansion will further provide a positive effect on proliferative advantage over the T-cell lineage. **(C)** When the germline mutation is deleterious for all hematopoietic lineages (i.e., the function of the affected gene is equally essential for all hematopoietic lineages), the reversion mutation in less-differentiated progenitor cells can reconstitute the whole hematopoietic system. In this scenario, somatic mosaicism can be detected in all compartments. Bone marrow failure disorders may be applicable to this model. HSC, hematopoietic stem cell; CLP, common lymphoid progenitor; CMP, common myeloid progenitor; GMP, granulocyte-monocyte progenitor; MEP, megakaryocyte-erythroid progenitor; NK, natural killer.

As observed in previous reversion cases in PIDs, reversion mutations are predominantly found in T-cell populations (**Tables 1**-**3**). This fact may reflect the consequence of the selective occurrence of reversion mutations in T-cell lineage. Extensive intrathymic cell division and the existence of self-renewing, long-persisting memory T-cells—that is, T_SCM_—may account for the selective reversion detection in T-cells (3, 89, 111). As an alternative, reversion mutations may occur in various cell lineages with the same probability or in less-differentiated hematopoietic progenitor cells. In this scenario, selective proliferation of revertant T-cells may be caused by any or all of the following factors: homeostatic expansion on the background of T-cell lymphopenia, intrinsic gene function for T-cell proliferation, and persistent antigen-specific responses (3) (**Figures 2A, B**). In the context of the basic pathology with bone marrow failure or neutropenia—that is, the function of an affected gene being equally essential for some or all hematopoietic lineages—reversion mutations have been detected in cells other than lymphoid lineages, as observed in SAMD9/SAMD9L syndrome, MYSM1 deficiency, WHIM syndrome, GATA2 deficiency, and Fanconi anemia (94, 96, 98, 105, 106, 112) (**Table 3** and **Figure 2C**). For the aforementioned reasons, reversion mutations may be predominant in T-cell populations.

The detection of somatic mosaicism by using conventional standard Sanger sequencing is challenging because the variant peak can be misinterpreted as background noise on the chromatogram when revertant cells constitute a minimal fraction (2). Highly sensitive and efficient molecular technologies such as deep sequencing technologies and quantitative techniques such as droplet digital PCR allow the detection and quantification of low-frequency mosaicism (1–3, 19, 113). These technologies can permit the efficient and accurate sequential observation of mosaicism, which will reveal important matters such as the relationship between spontaneous mosaicism oscillation and the clinical phenotype, the effect of alternative treatment on mosaicism [e.g., ADA deficiency (55–58) and XL-EDA-ID (71)], and the therapeutic effect of gene therapy (1). Furthermore, emerging sensitive and innovative methodologies, especially “single-cell” approaches, will facilitate reversion detection and the evaluation of characteristics, behavior, and fate of a revertant cell (3, 113).

Owing to overlapping and complicated phenotypes, the diagnosis of PIDs is not necessarily easy. Reversion mutations can modify clinical and immunological phenotypes; therefore, diagnostic delay and underdiagnosis can occur, particularly when the disease intrinsically presents with diverse clinical and immunological features, as seen in XL-EDA-ID (69).

Furthermore, the technical limitations and the nature of the gene may make a genetic diagnosis difficult. For example, transcriptomics is often used as a complementary diagnostic tool if patients remain without a molecular diagnosis after target next-generation sequencing, whole exome sequencing, and whole genome sequencing analysis; however, a molecular diagnosis can be delayed, if revertant wild-type allele is selectively amplified (84). In addition, if most blood cells are revertant cells, a molecular diagnosis can be hampered by the major detection of revertant wild-type alleles. Therefore, genetic analysis on DNA extracted from a tissue other than peripheral blood such as buccal mucosa cells and, optimally, skin fibroblasts may be advisable, particularly when a concern exists about a PID for which the reversion occurrence is relatively high such as WAS, DOCK8 deficiency, and XL-EDA-ID (3).

In summary, the clinical impact of somatic reversion depends on the type, differentiation, diversity, and number and function of cells in which reversion occurs, intrinsic function of an affected gene, and location of the germline mutation. In the context of the adaptation of gene therapy for PID patients, cells harboring artificial genomic changes should work as expected *in vivo*. To date, the mechanism of reversion and the function of revertant cells have been elucidated by means of emerging technologies, even in genes and in cell types in which reversion events have not been described. In the future, further technological evolutions will enable scientists to generate new perspectives for reversion mosaicism in PIDs.

## Author Contributions

HM and TW wrote and critically revised the manuscript. All authors contributed to the article and approved the submitted version.

## Conflict of Interest

The authors declare that the research was conducted in the absence of any commercial or financial relationships that could be construed as a potential conflict of interest.

The handling editor declared a past collaboration with one of the authors, TW.

## Publisher’s Note

All claims expressed in this article are solely those of the authors and do not necessarily represent those of their affiliated organizations, or those of the publisher, the editors and the reviewers. Any product that may be evaluated in this article, or claim that may be made by its manufacturer, is not guaranteed or endorsed by the publisher.
